# SII, SIRI, and MHR as Additional Readings for Personalized Evaluation of Chronic Heart Failure Severity

**DOI:** 10.3390/ijms26115190

**Published:** 2025-05-28

**Authors:** Edis Baubonis, Jolanta Laukaitienė, Ingrida Grabauskytė, Aušra Mongirdienė

**Affiliations:** 1 Medicine Academy, Lithuanian University of Health Sciences, Eiveniu Str. 4, LT-50103 Kaunas, Lithuania; edis.baubonis@stud.lsmu.lt; 2 Department of Biochemistry, Medicine Academy, Lithuanian University of Health Sciences, Eiveniu Str. 4, LT-50103 Kaunas, Lithuania; jolanta.laukaitiene@lsmu.lt; 3 Department of Physics, Mathematics and Biophysics, Medicine Academy, Lithuanian University of Health Sciences, Eiveniu Str. 4, LT-50103 Kaunas, Lithuania; ingrida.grabauskyte@lsmu.lt

**Keywords:** chronic heart failure, oxidative stress, inflammation, MHR, SII, SIRI

## Abstract

(1) The aim of the study was to reveal what differences in patients’ lipidogram, oxidative stress, and echocardiographic readings are reflected by SII, SIRI, and MHR of the patients with chronic heart failure (CHF). (2) A total of 220 patients diagnosed with CHF were investigated. They were stratified into groups according to averages of SII (neutrophil * platelet/lymphocyte count), SII ≤ 684.757 (*n* = 115), and SII > 684.757 (*n* = 62); SIRI (neutrophil * monocyte/lymphocyte count), SIRI ≤ 2.098 (*n* = 110), SIRI > 2.098 (*n* = 67); and monocyte count/high-density lipoprotein cholesterol concentration (MHR), MHR ≤ 0.5854 (*n* = 54), and MHR > 0.5854 (*n* = 64) values. The analysis of transthoracic echocardiogram, complete blood count test, C reactive protein, lipidogram, oxHDL, nitrotirozine, ditirozine, TAC, protein carbonyl, catalase, and MDA were performed; (3) Between the groups, according to SII and SIRI, there were no statistically significant differences in lipidogram, oxidative stress, and echocardiography readings. In those with higher MHR, HDL concentration was lower (0.91 (0.44; 1.45) and 1.27 (0.72; 2.69), *p* < 0.001). In those with higher MHR, LVEDD was higher (58.12 (10.03) and 51.53 (10.34), *p* < 0.001), LVMM was higher (274.92 (92.24) and 233.07 (74.84), *p* = 0.010), MMI was higher (130.88 (34.28; 227.97) and 114.27 (70.34; 270.00), *p* = 0.022), and LVEF was lower (28.5 (10.0; 55.0) and 40.0 (20.0; 55.0), *p* < 0.001). MHR correlated with MMI (r = 0.287, *p* = 0.028) and LVMI (r = 0.287, *p* = 0.028). Nitrotyrosine concentration was higher in those with higher MHR (4.52 (1.12; 93.58) and 3.52 (1.74; 28.32), *p* = 0.022). MHR correlated with protein carbonyl (r = 0.321, *p* = 0.013), nitrotyrosine concentration (r = 0.356, *p* = 0.006). SIRI correlated with carbonyl protein concentration (r = 0.321, *p* = 0.013); (4) 1. In CHF patients, MHR could reflect the worsening of patients’ conditions related to oxidative stress. 2. The possibility to use SII and SIRI still needs to be confirmed.

## 1. Introduction

Chronic heart failure (CHF) is a complex group of clinical syndromes. It is related to structural and functional abnormalities of the heart, resulting in patients’ deteriorating quality of life and death. Heart failure (HF) mortality and hospitalization rates have been increasing since 2012 despite the advances in the treatment [1]. So, searching for pathogenesis, and severity evaluation possibilities is still crucial to its early prevention.

Hypertension, obesity, and smoking are presented as increasing risk factors for HF [1]. All these risk factors are related to chronic inflammation, which is emphasized as a key pathway in the development of HF [2]. Inflammation contributes to the development of HF across the ejection fraction (EF) spectrum, and HF itself promotes a chronic inflammatory state [3]. Oxidative stress and inflammation are interdependent conditions that occur simultaneously. Oxidative stress products enhance pro-inflammatory responses, whereas inflammatory cells release ROS at the site of inflammation and promote oxidative damage. Oxidative stress and inflammatory biomarkers are thought to be promising diagnostic and prognostic tools in patients with HF [4]. In recent years, total blood count-based readings of neutrophil–lymphocyte ratio (NLR) [5], lymphocyte–monocyte ratio (LMR) [6], platelet–lymphocyte ratio (PLR) [6], systemic inflammation index (SII, neutrophil * platelet /lymphocyte count), systemic inflammatory response index (SIRI, neutrophil * monocyte/lymphocyte count) [7], and MHR (monocyte count/high-density lipoprotein cholesterol concentration) [8] have been investigated to assess the severity and the prognosis of patients with CHF and to avoid patient hospitalization. 

C. Delcea and coauthors reviewed the role of NLR in HF, including CHF, and concluded that increased NLR can be a valuable indicator of HF severity and poor prognosis [9]. M. Vakhshoori and coauthors reviewed that an increase in both LMR and MLR was associated with long-term mortality in HF patients [10]. An epidemiological study also revealed that considering monocytes and high-density lipoprotein cholesterol (HDL-C) concurrently can better predict coronary arteriosclerosis [11]. But in this review, there was no detailed information about the HF forms investigated. Additionally, LMR and MHR were found to be complementary markers of the CHF diagnosis [8]. It is obvious that total blood count-based readings could not be used in acute inflammatory states among HF patients. As novel, simply measurable inflammation readings, SII and SIRI are not well characterized in CHF patients yet. They were found to be associated with increased risk of cardiovascular diseases (CVDs) [12]. Also, SII was observed to predict adverse outcomes in CVD patients [13,14]. SII and SIRI could be appropriate for evaluation of inflammatory state, because these readings involve monocytes, lymphocytes, and neutrophils. These cells are mostly related to inflammation. MHR involves HDL-C and monocytes, so could show both inflammation and lipid transport influence on the CHF condition. The correlations between inflammation markers and HF have been widely discussed in recent years [2,15], but the role of systemic inflammatory indicators (such as SII, SIRI, and MHR) in CHF has not yet been established. 

It is known that chronic CHF can occur with a reduced or preserved left ventricle ejection fraction (LVEF), and HFrEF can occur after MI or without MI history. HFrEF can be the result of MI, and HFpEF can be the result of concomitant diseases (overweight/obesity, diabetes mellitus, dyslipidemia) [16,17]. HFpEF can change to HFrEF. So, we aimed to reveal what differences in patients’ lipidograms, oxidative stress levels, and echocardiographic readings are reflected by SII, SIRI, and MHR. These differences should show whether chronic inflammation readings SII, SIRI, and MHR could be useful as additional readings for specifying the condition of patients with CHF. The differences should also be helpful for determining the readings’ eligibility for use as additional markers for evaluation of CHF patients’ proinflammatory condition and specifying the blood cells that are most involved in supporting chronic inflammation and which could be used for the treatment targets search. Additionally, the importance of personalizing treatment has been emphasized depending on the specific condition. 

## 2. Results

### 2.1. Readings’ Differences Between the Groups According to SII

PLT, leukocyte, and neutrophil counts were higher, and the lymphocyte count was lower in those with higher SII (Table 1). NLR and SIRI were higher in those with higher SII (Table 2). Lipid concentration does not differ in the groups according to SII, as well as readings of oxidative stress and echocardiography.

### 2.2. Differences in Laboratory and Echocardiography Readings Between the Groups According to MHR

Monocyte count, log SIRI, and log SII were higher in those with higher MHR (Table 3). In the group with higher MHR, cholesterol, LDL, and HDL concentrations were lower (Table 4). Oxidative stress readings did not differ between the groups, except for MDA and nitrotyrosine. MDA was lower and nitrotyrosine was higher in the group with higher MHR (Table 5). KSGDD L VEDD and MMI LVM I were higher in those with higher MHR (Table 6).

### 2.3. Comparison of Laboratory and Echocardiography Readings Between the Groups According to SIRI

Leukocyte, neutrophil, and monocyte counts were higher, and lymphocyte counts were lower in the groups with higher SIRI. Accordingly, NLR and SII readings in this group were higher too (Table 7). Lipidogram, oxidative stress, and echocardiogram readings did not differ. 

## 3. Discussion

Our work revealed interesting findings that between the groups, according to SII and SIRI, there were no statistically significant differences in lipidogram, oxidative stress concentration, and exocardiography readings. But in groups according to MHR, some differences were found. Those with higher MHR were more likely to be male and to have a higher BMI (Table 1). Accordingly, despite the lower concentrations of total and LDL cholesterol in the group with higher MHR, HDL cholesterol concentration in these patients was lower too, and the index of atherogenicity was higher (Table 5). MHR was the only reading in which echocardiographic readings differed between the groups (Table 7). Accordingly, MHR correlated with some echocardiographic readings. In oxidative stress readings, nitrotyrosine concentration was statistically significantly higher in those with higher MHR. MHR correlated with oxidative stress readings, nitrotyrosine, and protein carbonyl concentration as well. SIRI correlated with protein carbonyl concentration too.

The correlations between inflammation markers and HF have been widely discussed in recent years [2]. But the role of systemic inflammatory indicators (such as SII, SIRI and MHR) in CHF has not yet been established.

### 3.1. Systemic Inflammation and Its Relationship with HDL in CHF

It was reported that the myocardial injury of patients with HF would activate the immune system, which is the trigger of the systemic inflammatory state [18]. Accordingly, HFrEF was found to be associated with elevated circulating levels of pro-inflammatory cytokines [3]. Monocyte and macrophage activation and polarization are initiated through the recognition of pathogen- and tissue damage-associated conserved molecular motifs through pattern recognition receptors [19]. In monocytes and macrophages, this pathway triggers the transcription of inflammatory cytokines such as TNF-α, IL-6, and IL-1β. These cytokines promote the resulting immune response and induce the prolonged survival of monocytes [20]. Chronic inflammation is characterized by a continuous recruitment of monocytes and lymphocytes [21]. Monocytes and macrophages are key cellular components involved in the development and regulation of numerous chronic inflammatory conditions [21]. An elevated monocyte count is known to signify an inflammatory state. Monocytes participate in the onset and the resolution phases of inflammation. During inflammation, monocytes can differentiate into tissue macrophages that significantly enhance the inflammatory response and secrete cytokines that help combat infection and facilitate the resolution of inflammation [21]. 

Dividing the patients into groups according to MHR in our study indicates that the patients with higher monocyte count and lower HDL have worse cardiac function, higher BMI, and higher oxidative stress. So, the MHR could reflect the worse patient’s condition.

Accordingly, it has been demonstrated that HDL exerts a protective role in atherosclerosis by inhibiting the expression of endothelial adhesion molecules. This action reduces the recruitment and accumulation of monocytes at sites of vascular injury. By preventing monocyte adhesion to the endothelial cells, HDL helps to mitigate the inflammatory processes that contribute to the formation and progression of atherosclerotic plaques [22]. It was found that MHR, consisting of monocytes, an inflammatory marker, and HDL, an antiatherogenic lipid parameter, was associated with a high SYNTAX score in patients with stable coronary artery disease (CAD) [23]. There are studies showing that HDL is effective in monocyte activation and inflammation in the development of atherosclerosis [24,25,26]. It was revealed that low concentrations of HDL cholesterol have been associated with a higher incidence of HF [27]. Impaired HDL function in CHF patients leads to a higher pro-inflammation status in these patients [28]. 

HDL-C can help to reduce inflammation by transporting cholesterol away from the arteries and inhibiting the oxidation of low-density lipoprotein [29]. Accordingly, HDL-C can inhibit the expression of endothelial adhesion molecules, thereby reducing monocyte aggregation [30]. In persons with higher BMI, the count of non-classical monocyte subgroups is higher [31]. Our results supplement and fit these findings.

Only a few studies have explored the associations between MHR and HF, as its components, monocytes and HDL-C, have been broadly discussed in HF [28,32]. MHR has been found to be increased in patients with more serious CHF (*p* = 0.001) and was associated with CHF [8]. Our findings showed the same. In those with higher MHR, we found higher BMI, lower HDL and LVEF, and higher left ventricular end-diastolic diameter (LVEDD), left ventricular myocardial mass (LVM), and myocardium mass index (MMI). Accordingly, our results show that MHR correlated with these heart ultrasound readings. It is known that increased LVEDD, LVMM, and LVMI could show structural changes in the heart, such as myocardial hypertrophy and HF. It should be mentioned that after Bonferroni correction, LVMM and LVMI differences between the groups according to MHR disappeared, but differences in LVEF and LVEDD remained. So, our findings revealed a statistically significant correlation between MHR and readings of structural changes in the heart. These findings could demonstrate that inflammation plays an important role in CHF, and the inflammatory indicator MHR could be a complementary biomarker in the evaluation of the statement of CHF. Additionally, the results of anti-inflammatory therapy targeting these markers might be promising. It is worth mentioning that interleukin-1β inhibition with canakinumab markedly reduced plasma levels of interleukin-6 and high-sensitivity C-reactive protein without lowering the level of low-density lipoprotein (LDL) cholesterol [33].

It was determined that patients in the HFrEF group presented higher MHR values than the HFpEF group (*p* = 0.001), and lower MHR values in the 1st tertile group than the 2nd tertile group (*p* = 0.008) and the 3rd tertile group (*p* = 0.002), which indicates that the level of MHR was associated with the severity of CHF [8]. Our findings supplement these results because we found LVEDD, LVM, and MMI to be higher in those with higher MHR (Table 6). Furthermore, MHR correlated with these exocardiographic readings. 

Summarizing the discussed findings, it could be stated that (1) MHR can reflect the worst patient’s condition in CHF patients and (2) can justify the readings‘ relationship with the chronic inflammation.

### 3.2. Interfaces Between Systemic Inflammation and Oxidative Stress Readings in CHF 

The inflammatory process induces oxidative stress, which is characterized by high levels of ROS overwhelming the buffering capacity of the antioxidant system [21]. Thus, inflammation and oxidative stress are closely intertwined; inflammatory cells release ROS at the site of inflammation and promote oxidative damage, whereas oxidative stress products enhance pro-inflammatory responses [34]. We found a higher concentration of nitrotyrosine in oxidative stress readings in those with higher MHR (as an inflammatory reading). Accordingly, MHR correlated both with nitrotyrosine and protein carbonyl concentration. Also, it is stated that the right ventricle (RV) exhibits heightened vulnerability to oxidative stress in comparison to the left ventricle (LV). This increased susceptibility in the RV is partly attributed to its inability to regulate the expression of manganese superoxide dismutase, which contributes to oxidative stress in the RV [35]. Our findings fit these results, because we revealed that those with higher MHR had higher left ventricle parameters LVEDD, LVMI and LVM, reflecting worse cardiac function.

Another oxidative stress reading, MDA, is presented as used by the researchers to evaluate the extent of lipid peroxidation, which is a process where cell membrane lipids are damaged by oxidative stress, leading to the formation of MDA as a byproduct [36]. Some results suggest that MDA may play a crucial role in the pathophysiology of HFrEF that is independently related to poor prognosis [36,37]. In HFrEF patients with ischemic cardiomyopathy (ICM) (endpoint (EP)+), higher MDA concentrations than those in patients with ICM (EP−) were indicated. Similarly, MDA concentrations were higher in the nICM (EP+) than in the nICM (EP−) group. Additionally, TAC was the most increased in nICM (EP+) [36]. Our results obtained in HFrEF patients show MDA is lower in the higher MHR group and no correlations between MDA and inflammation, exocardiographic, and other oxidative stress parameters or lipid concentration were found. TAC does not differ statistically significantly between MHR groups. These differences may be due to patients‘ differences: our patients have such diagnoses according to the International Classification of Diseases (ICD-10), as follows: I50.0—congestive heart failure (left ventricular failure), I50.1—congestive heart failure (right ventricular failure), I11.0—hypertensive heart disease (with heart failure), so the pathogenesis of developed heart failure in our patients was not the same despite the fact that all of them had CHF. Additionally, adjusted α by Bonferroni correction was α = 0.05/8 = 0.006. So, the difference in MDA between the groups could be statistically insignificant according to Bonferroni correction. A previous study has shown that oxidative stress is related to the severity of heart failure, although the results were different in patients with ischemic and nonischemic cardiomyopathy [38]. 

Oxidative and nitrosative stress are thought to be able to trigger immune responses, exacerbating inflammation and contributing to cardiovascular disease progression [39]. Elevated circulating nitrotirosine (3-NT) levels are associated with increased fibrosis and structural remodelling of the myocardium, highlighting their potential role as a diagnostic marker and therapeutic target [40]. It was found that nitrotyrosine negatively correlated with HDL cholesterol (r = −0.46; *p* < 0.05) in cardiovascular disease patients [41]. In Karol Momot’s research, myeloperoxidase (MPO—enzyme that takes part in the production of nitrotyrosine) concentrations do not differ between the HFrEF (related to inflammation), HFpEF (related to fibrosis) and control groups [40]. Another study, which compared biomarkers between HFpEF and HFrEF, showed that circulating levels of MPO were similar in both groups [42]. We found that nitrotyrosine concentration correlated with MHR and was higher in those with higher MHR in our HFrEF patients. Accordingly, nitrotyrosine concentration correlated with other oxidative stress readings, such as protein carbonyl concentration and catalase activity (negative correlation), in our study. In the other study, body mass index (BMI) positively correlated with MPO among the HFpEF population [12]. In our research, both nitrotyrosine protein carbonyl concentration and BMI correlated with MHR and were higher in those with higher MHR. Other oxidative stress readings did not differ between the groups according to MHR and did not correlate with MHR or BMI. It should be mentioned that after adjusting α by Bonferroni correction, the statistical significance disappeared. Despite this, correlations between nitrotyrosine concentration and MHR (what we have found) suggest a link between them and confirm the difference we found between the groups.

In conclusion, it could be stated that nitrotyrosine concentration has the strongest relationship with chronic inflammation in our investigated CHF patients, and this finding supports the results of other studies’ results. It should be mentioned that our results did not show a relationship between the other oxidative stress readings, such as TAC, MDA, OxDTL, and the MHR in CHF patients. 

### 3.3. Eligibility of Grouping by SIRI and SII for Finding Lipid, Oxidative Stress, and Echocardiogram Readings‘ Differences in CHF Patients

SIRI and SII, calculated by the neutrophil, lymphocyte, monocyte, and platelet counts, have been previously reported to reflect the systemic inflammatory response of the body [43]. Monocytes are presented as major drivers of inflammatory and fibrotic processes in heart diseases and HF [44]. Neutrophils could secrete inflammation mediators, chemotactic agents, and oxygen-free radicals to induce endothelial cell injury and subsequent tissue ischemia [5,7]. On the contrary, lymphocytes have a regulatory function in inflammation [7]. Both SII and SIRI are thought to be useful for prognosis in CHF patients [5,6,7,8,9,12,14]. It seemed the chronic inflammation readings, such as SII, SIRI, and MHR, could be proper for clarifying chronic inflammation and the condition in CHF patients, as reflected by oxidative stress and echocardiogram parameters. Kevser B. and coauthors found that a higher SII was associated with right ventricle dysfunction and lower LVEF in HFrEF [45]. Wojciechowska et al. showed a relationship between inflammation and systemic and pulmonary hemodynamic parameters; serum activities of superoxide dismutase isoenzymes significantly correlated with pulmonary capillary wedge pressure, mean pulmonary artery pressure, and LVEF in patients with dilated cardiomyopathy [46]. SII was also identified as an independent risk factor for non-left ventricle reverse remodeling (LVRR) and was predictive of poor prognosis in patients with HFrEF [12]. These findings could indicate that indirect inflammation parameters could be informative regarding hemodynamic status. But we found no differences between the groups according to SII and SIRI. Only SIRI correlated with protein carbonyl concentration. The reasons for those discrepancies could be because of patients’ comorbidities, different groupings, or different study designs used. Accordingly, it seems it would be more precise to first investigate HFrEF and HFpEF patients without any comorbidities separately. Our patients have LVEF ≤ 55%, so our population includes HFrEF and HFpEF patients (those who had LVEF 50–55%). 

The results of our study support the effort to highlight the assessment of MHR, SIRI, and SII in policies for CHF and provide further therapeutic targets to improve the prognosis. The management of SIRI and SII and its beneficial effect on the CHF condition reflection deserves further exploration. In contrast, MHR seems to be related to CHF patients’ conditions and could be used as additional reading after establishing thresholds for each specific group of patients.

## 4. Materials and Methods

### 4.1. Study Population

A total of 220 patients admitted to the Department of Cardiology of Kaunas Clinical Hospital of Lithuanian University of Health Sciences between 1 January 2016 and 1 March 2018 (*n* = 60) and diagnosed with CHF (left ventricle ejection fraction (LVEF) ≤ 55%) were included in the study. All the patients gave written consent. The patients were hospitalized for heart rhythm and other non-infectious reasons.

Additionally, the data from 1 January 2018 to 1 February 2021 were collected (*n* = 160) from the Hospital of Lithuanian University of Health Sciences Kauno Klinikos Cardiology Department and evaluated retrospectively. Inclusion criteria for patients retrospectively selected and admitted to the hospital were no changes in functional class according to the New York Heart Association (NYHA) and no changes in treatment with medicines within the past 4 weeks. Moreover, the included patients did not have any new HF symptoms and had LVEF ≤ 55%. The diagnosis of CHF was performed by following the guidelines for the diagnostics and treatment of heart failure approved by the European Society of Cardiology [16]. Exclusion criteria were patients with kidney failure (eGFG < 60 mL/min), acute or chronic infection, acute coronary syndromes, diabetes mellitus, connective tissue disease, and smoking. All the investigations were approved and conducted in accordance with the guidelines of the local Bioethics Committee and adhered to the principles of the Declaration of Helsinki and Title 45, U.S. Code of Federal Regulations, Part 46, Protection of Human Subjects (revised 15 January 2009, effective 14 July 2009). The study was approved by the Regional Bioethics Committee at the Lithuanian University of Health Sciences (No. BE-2-102, 20 December 2018 and No. BE-2-2, 12 February 2020).

All the patients were stratified into groups according to averages of SII (SII ≤ 684.757 (*n* = 115) and SII > 684.757 (*n* = 62), SIRI (SIRI ≤ 2.098 (*n* = 110) and SIRI > 2.098 (*n* = 67)), and MHR (MHR ≤ 0.5854 (*n* = 54) and MHR > 0.5854 (*n* = 64)) values. According to the New York Heart Association (NYHA) classification of severity of heart failure, there was an equal distribution of patients in NYHA classification categories (Class II–IV) between the groups. The sociodemographic and clinical characteristics between the groups based on SII and SIRI did not differ. The patients in the group with lower MHR were older, had lower body mass index (BMI), and higher left ventricle ejection fraction (LVEF) (Table 8). Drug usage between the groups, according to MHR, did not differ.

### 4.2. Investigated Readings and Methods

The analysis of transthoracic echocardiogram, complete blood count test, C reactive protein, lipidogram, oxidative stress readings (oxidized high-density lipoprotein (oxHDL)), nitrotirozine, ditirozine, total antioxidant capacity (TAC), protein carbonyl, catalase, and malondialdehyde acid (MDA) were performed after patients’ admission to the hospital. For the tests, blood samples of fasting patients were taken from the forearm vein and processed as usual for complete blood count, C reactive protein, and lipidogram. OxHDL, nitrotirozine, ditirozine, TAC, protein carbonyl, catalase and MDA were investigated in blood serum, which was frozen at −80 °C until analysis. 

Oxidative and antioxidative markers were measured in serum using the following commercial reagent kits: human total antioxidant capacity ELISA Kit abx053643 (abbexa, Cambridge, UK), carbonyl protein ELISA K7870 (immune diagnostic AG), human oxidized high-density lipoprotein (Ox-HDL0 ELISA Kit CSB-E16552h (Cusabio biotech Co., Wuhan, China), and nitrotyrosine ELISA K7829 (immune diagnostic AG). Catalase (CAT) activity in serum was evaluated according to the method described in [47]. CAT activity was measured by hydrogen peroxide reaction with ammonium molybdate, which produces a complex that absorbs at a wavelength of light of 410 nm. The results were expressed in U/mg protein. Under these conditions, one unit of catalase (U) decomposes 1 _mol of hydrogen peroxide per 1 min. The protein concentration in serum was measured using the Lowry method [48]. Samples for MDA were prepared and analyzed according to the methodology of Khoschosorur et al. [47] using the HPLC method with fluorescence detection. Chromatographic separation was performed on the HPLC system (Shimadzu Nexera X2). A 20-μL sample was injected on the HPLC column (Agilent Poroshell 120 EC–C18, 3 × 100 mm, 2.7 μm). The chromatographic isocratic separation was carried out with a binary mobile phase of methanol and 50 mM phosphate buffer, pH 6.8 (2:3, *v*/*v*). Fluorescence detection was performed at 230 nm excitation and 430 nm emission wavelengths. The average retention time of the malondialdehyde–thiobarbituric acid adduct was 1.63 min.

### 4.3. Statistical Analysis

Microsoft Office Excel and IBM SPSS Statistics version 29.0 were used for data analysis. To test the normality assumption, the data was assessed with the Kolmogorov–Smirnov and Shapiro–Wilk tests. Two groups were compared by an independent samples *t*-test (normality assumption satisfied). For non-parametric statistics, a Mann–Whitney U test was performed for comparison between the two groups (normality assumption not satisfied). Qualitative variables are compared by the Chi-square test. Pearson’s correlation (rp) analysis was performed when two variables were normally distributed; otherwise, Spearman’s correlation (rs) analysis was used. Differences when the *p*-value was less than 0.05 were considered statistically significant.

## 5. Limitations

First, the patients of our study were only enrolled from Lithuania; thus, the findings of our study may not be completely generalized to other regions. Second, not all patients had oxidative stress and lipidogram results. Third, our patients were not precisely homogenous; they had diagnoses of congestive heart failure (left ventricular failure), congestive heart failure (right ventricular failure), and hypertensive heart disease (with heart failure). So, investigating the results obtained from the patients with one diagnosis will better reveal our chosen chronic inflammatory readings‘ eligibility for reflecting specific patient conditions. Fourth, other inflammation and heart fibrosis readings would be welcome for a wider evaluation of the patient’s statement. Fifth, the absence of a multivariable adjustment for confounders. Sixth, the cross-sectional design limiting causal inference, and lack of longitudinal outcomes (e.g., mortality, rehospitalization). 

## 6. Conclusions

(1) MHR could reflect the worst patients’ condition (dependent on echocardiography results) in CHF patients. (2) The possibility to use SII and SIRI as additional readings for determining the condition of the patient with CHF still needs to be confirmed in future randomized studies.

## Figures and Tables

**Table 1:** Results of total blood count in the groups according to SII.

	SII ≤ 684.757 (*n* = 115)	SII > 684.757 (*n* = 62)	*p* Value
PLT, 10^9^/L	192 (155–222)	226 (–252)	<0.001
MPV, Fl	10.5 (9.6–11.2)	10.5 (9.6–11.3)	0.622
LEU, 10^9^/L	6.28 (5.30–7.59)	7.30 (6.46–9.01)	<0.001
NEUTR, 10^9^/L	3.582 (2.932–4.527)	4.973 (4.236–6.296)	<0.001
LYMPH, 10^9^/L	1.714 (1.409–2.376)	1.264 (1.045–1.587)	<0.001
MONO, 10^9^/L	0.632 (0.494–0.829)	0.676 (0.584–0.900)	0.087

PLT—platelet count, MPV—mean platelet volume, LEU—leukocyte count, NEUTR—neutrophil count, LYMPH—lymphocyte count, MONO—monocyte count. The median (IQR) of the results is given. A non-parametric Mann–Whitney U test was applied to compare quantitative variables between the two groups. Adjusted α by Bonferroni correction—α = 0.05/6 = 0.008.

**Table 2:** Calculated readings of total blood count in the groups according to SII.

	SII ≤ 684.757 (*n* = 115)	SII > 684.757 (*n* = 62)	*p* Value
NLR, Total	1.970 (1.565–2.609) 115	3.680 (3.438–4.775) 62	<0.001
Log NLR, Total	0.47 (0.41–0.56) 115	0.67 (0.65–0.76) 62	<0.001
SIRI, Total	12.49 (9.40–18.0) 115	27.35 (21.45–37.38) 62	<0.001
Log SIRI, Total	0.35 (0.29–0.45) 115	0.57 (0.50–0.68) 62	<0.001
MHR Total	0.62 (0.46–0.79) 69	0.62 (0.44–0.88) 49	0.546
Log MHR Total	0.21 (0.17–0.25) 69	0.21 (0.16–0.28) 49	0.562

NLR—neutrophil-lymphocyte ratio, SIRI—neutrophil * monocyte/lymphocyte count, MHR—monocyte count/high-density lipoprotein cholesterol concentration. The median (IQR) of the results is given. A non-parametric Mann–Whitney U test was applied to compare quantitative variables between the two groups. Adjusted α by Bonferroni correction—α = 0.05/6 = 0.008.

**Table 3:** Results of total blood count in the groups according to MHR average.

	MHR_average ≤ 0.5854 (*n* = 54)	MHR_average > 0.5854 (*n* = 64)	*p* Value
PLT, 10^9^/L	204.5 (167.0–236.0)	205.0 (169.5–235.3)	0.768
MPV, Fl	10.5 (9.9–11.5)	10.7 (9.7–11.4)	0.918
LEU, 10^9^/L	6.690 (5.375–7.540)	6.885 (5.800–8.605)	0.110
NEUTR, 10^9^/L	4.096 (3.104–4.969)	4.470 (3.391–5.437)	0.226
LYMPH 10^9^/L	1.435 (1.182–2.140)	1.550 (1.285–2.013)	0.292
MONO 10^9^/L	0.559 (0.461–0.641)	0.763 (0.629–0.933)	<0.001
NLR 10^9^/L	2.82 (1.80–3.71)	2.66 (1.90–3.56)	0.966
* Log NLR	0.585 (0.168)	0.579 (0.143)	0.856
SIRI	1.47 (0.96–2.25)	2.11 (1.38–3.22)	0.002
Log SIRI	0.395 (0.290–0.510)	0.490 (0.375–0.623)	0.001
SII	580.67 (354.43–794.26)	522.20 (362.43–803.54)	0.837
Log SII	0.395 (0.290–0.510)	0.49 (0.375–0.623)	0.001

PLT—platelet count, MPV—mean platelet volume, LEU—leukocyte count, NEUTR—neutrophil count, LYMPH—lymphocyte count, MONO—monocyte count, NLR—neutrophil–lymphocyte ratio. The median (IQR) (non-parametric Mann–Whitney U test was applied to compare quantitative variables between the two groups), and *—mean (std. deviation) (Student *t* test was applied to compare quantitative variables between the two groups) of the results are given. Adjusted α by Bonferroni correction—α = 0.05/12 = 0.004.

**Table 4:** Results of lipid concentration in the groups according to MHR average.

	MHR_average ≤ 0.5854	MHR_average > 0.5854	*p* Value
Cholesterol, g/L Total	5.27 (4.11–6.05) 53	4.02 (3.41–4.85) 64	<0.001
LDLch, Total	3.36 (2.52–3.98) 54	2.66 (2.12–3.31) 64	0.008
HDLch, Total	1.27 (1.10–1.59) 54	0.91 (0.77–1.05) 64	<0.001
TAG, Total	1.26 (0.74–1.69) 54	1.21 (0.83–1.65) 64	0.746
Atherogenicity coeff., Total	2.44 (2.18–3.57) 49	3.53 (2.90–4.60) 61	<0.001

LDL—low-density lipoprotein, HDL—high-density lipoprotein, TAG—triacylglycerol. The median (IQR) of the results is given. A non-parametric Mann–Whitney U test was applied to compare quantitative variables between the two groups. Adjusted α by Bonferroni correction—α = 0.05/5 = 0.010.

**Table 5:** Results of oxidative stress readings in the groups according to MHR average.

	MHR_average ≤ 0.5854 (*n* = 29)	MHR_average > 0.5854 (*n* = 30)	*p* Value
oxDTL, pg/mL	3.109 (2.229–8.532)	3.069 (2.176–4.583)	0.596
Nitrotyrosine, nM	3.517 (2.640–4.407)	4.523 (3.199–6.301)	0.022
Dityrosine, RUF	7.62 (6.57–8.77)	8.08 (7.25–9.40)	0.197
TAC, U/mL	0.63 (0.31–0.92)	0.57 (0.21–1.84)	0.549
Protein carbonyl, U/mL	244.90 (196.00–285.76)	291.47 (288.79–379.73)	0.051
Catalase, U/mg	2.00 (1.65–3.01)	2.02 (1.52–2.72)	0.733
Catalase, U/mL	132.97 (103.42–182.47)	132.99 (103.42–177.30)	0.767
MDA, µg/L	119.62 (110.93–134.13)	107.10 (89.20–126.33)	0.026

oxDTL—oxidized low-density lipoprotein, TAC—total antioxidant capacity, MDA—malondialdehyde acid, relative units of fluorescence. The median (IQR) of the results is given. A non-parametric Mann–Whitney U test was applied to compare quantitative variables between the two groups. Adjusted α by Bonferroni correction—α = 0.05/8 = 0.006.

**Table 6:** Echocardiography results in the groups according to MHR average.

	MHR_average ≤ 0.5854	MHR_average > 0.5854	*p* Value
LVEF, % Total	40.0 (25.0–46.0) 54	28.5 (20.0–40.0) 64	<0.001
* L VEDD, mm Total	51.53 (10.34) 51	58.12 (10.03) 62	<0.001
SSI_R_WT_cm, Total	0.44 (0.39–0.51) 29	0.38 (0.32–0.48) 30	0.051
* L_VMM_g, Total	233.07 (74.835) 51	274.92 (92.239) 62	0.010
LVM_I_g/m^2^, Total	112.74 (91.83–124.74) 29	130.15 (103.14–162.79) 30	0.022
KPP_m^2^, Total	1.85 (1.75–2.03) 29	1.98 (1.82–2.18) 30	0.068
LVMI, g/m^2^ Total	112.74 (91.83–124.74) 29	130.15 (103.24–162.79) 30	0.058

LVEF—left ventricle ejection fraction, L VEDD—left ventricular end-diastolic diameter, SSI_R_WT—systolic strain index of right ventricular wall thickness, L_VMM—left ventricular myocardial mass, LVM_I—myocardial mass index, KPP—cardiac power production, LVMI—left ventricle mass index. The median (IQR) (non-parametric Mann–Whitney U test was applied to compare quantitative variables between the two groups), and *—mean (std. deviation) (Student *t* test was applied to compare quantitative variables between the two groups) of the results are given. Adjusted α by Bonferroni correction—α = 0.05/7 = 0.007.

**Table 7:** Results of total blood count in the groups according to SIRI average.

	SIRI average ≤ 2.098 (*n* = 110)	SIRI average > 2.098 (*n* = 67)	*p* Value
PLT, ×10^9^/L	197.0 (158.5–233.3)	203.0 (190.0–247.0)	0.034
MPV, Fl	10.6 (9.6–11.2)	10.4 (9.3–11.4)	0.905
LEU, ×10^9^/L	6.10 (5.21–7.33)	7.60 (6.50–8.91)	<0.001
NEUTR, ×10^9^/L	3.48 (2.90–4.38)	4.98 (4.26–6.09)	<0.001
LYMPH, ×10^9^/L	1.71 (1.38–2.38)	1.35 (1.05–1.70)	<0.001
* MONO, ×10^9^/L	0.61 (0.200)	0.85 (0.265)	<0.001
NLR	1.93 (1.56–2.58)	3.63 (3.25–4.75)	<0.001
Log NLR	0.47 (0.41–0.55)	0.67 (0.63–0.76)	<0.001
SII	369.73 (286.33–508.87)	790.00 (681.52–962.43)	<0.001
Log SII	0.34 (0.28–0.42)	0.59 (0.53–0.69)	<0.001

PLT—platelet count, MPV—mean platelet volume, LEU—leukocyte count, NEUTR—neutrophil count, LYMPH—lymphocyte count, MONO—monocyte count, NLR—neutrophil–lymphocyte ratio. The median (IQR) (non-parametric Mann–Whitney U test was applied to compare quantitative variables between the two groups), and *—mean (std. deviation) (Student *t* test was applied to compare quantitative variables between the two groups) of the results are given. Adjusted α by Bonferroni correction—α = 0.05/10 = 0.005.

**Table 8:** Baseline characteristics of groups according to the MHR average.

	MHR_average ≤ 0.5854	MHR_average > 0.5854	*p* Value
* Age, years Total	67.69 (13.368) 54	62.34 (14.947) 64	0.045
Gender, *n* (%)			0.009
Male	29 (53.7)	49 (76.6)	
Female,	25 (46.3)	15 (23.4)	
Total	54	64	
BMI, kg/m^2^	26.23 (24.02–29.28)	30.42 (25.31–34.16)	0.029
Total	41	53	
Systolic BP, mmHG	136.0 (123.5–141.0)	130.0 (118.0–141.5)	0.278
Total	54	64	
Diastolic BP, mmHg	80.5 (73.0–90.0)	80.0 (70.0–89.8)	0.470
Total	54	64	
Ischaemic heart disease, *n* (%)			0.099
0	31 (57.4)	27 (42.2)	
1	23 (42.6)	37 (57.8)	
Total	54	64	
PV_PP, *n* (%)			0.525
1	23 (42.6)	31 (48.4)	
2	31 (57.4)	33 (51.6)	
Total	54	64	

BMI—body mass index, BP—blood pressure, PP_PV—atrial flutter/atrial fibrillation. The median (IQR) (non-parametric Mann–Whitney U test was applied to compare quantitative variables between the two groups), and *—mean (std. deviation) (Student *t* test was applied to compare quantitative variables between the two groups) of the results are given. Qualitative variables are compared by the Chi-square test. Adjusted α by Bonferroni correction—α = 0.05/7 = 0.007.

## Data Availability

It is not possible to share the data according to guidelines of the local Bioethics Committee, who granted permission to conduct the research.

## Abbreviations

The following abbreviations are used in this manuscript:

CHF	chronic heart failure
SII	systemic inflammation index
SIRI	systemic inflammatory response index
MHR	monocyte count/high-density lipoprotein cholesterol concentration
